# Individual‐specific variation in the respiratory activities of HMECs and their bioenergetic response to IGF1 and TNFα

**DOI:** 10.1002/jcp.25932

**Published:** 2017-05-15

**Authors:** Sallie S. Schneider, Elizabeth M. Henchey, Nazneen Sultana, Stephanie M. Morin, D. Joseph Jerry, Grace Makari‐Judson, Giovanna M. Crisi, Richard B. Arenas, Melissa Johnson, Holly S. Mason, Nagendra Yadava

**Affiliations:** ^1^ Pioneer Valley Life Sciences Institute (PVLSI) Springfield Massachusetts; ^2^ Department of Veterinary & Animal Sciences University of Massachusetts Amherst Massachusetts; ^3^ Division of Hematology Oncology Department of Medicine at Baystate Medical Center/Tufts University School of Medicine Springfield Massachusetts; ^4^ Division of Anatomic and Clinical Pathology Department of Pathology at University of Massachusetts Medical School (UMMS)‐Baystate Regional Campus Springfield Massachusetts; ^5^ Division of Surgical Oncology Department of Surgery at University of Massachusetts Medical School (UMMS)‐Baystate Regional Campus Springfield Massachusetts; ^6^ Pioneer Valley Plastic Surgery Springfield Massachusetts; ^7^ Divisions of Endocrinology, Diabetes and Metabolism Department of Medicine at Baystate Medical Center /Tufts University School of Medicine Springfield Massachusetts; ^8^ Department of Biology University of Massachusetts Amherst Massachusetts

**Keywords:** bioenergetics, human mammary epithelial cells (HMECs), IGF1, oxidative phosphorylation, respiration, respiratory chain, TNFα

## Abstract

Metabolic reprograming is a hallmark of cancer cells. However, the roles of pre‐existing differences in normal cells metabolism toward cancer risk is not known. In order to assess pre‐existing variations in normal cell metabolism, we have quantified the inter‐individual variation in oxidative metabolism of normal primary human mammary epithelial cells (HMECs). We then assessed their response to selected cytokines such as insulin growth factor 1 (IGF1) and tumor necrosis factor alpha (TNFα), which are associated with breast cancer risk. Specifically, we compared the oxidative metabolism of HMECs obtained from women with breast cancer and without cancer. Our data show considerable inter‐individual variation in respiratory activities of HMECs from different women. A bioenergetic parameter called pyruvate‐stimulated respiration (PySR) was identified as a key distinguishing feature of HMECs from women with breast cancer and without cancer. Samples showing PySR over 20% of basal respiration rate were considered PySR^+ve^ and the rest as PySR^−ve^. By this criterion, HMECs from tumor‐affected breasts (AB) and non‐tumor affected breasts (NAB) of cancer patients were mostly PySR^−ve^ (88% and 89%, respectively), while HMECs from non‐cancer patients were mostly PySR^+ve^ (57%). This suggests that PySR^−ve/+ve^ phenotypes are individual‐specific and are not caused by field effects due to the presence of tumor. The effects of IGF1 and TNFα treatments on HMECs revealed that both suppressed respiration and extracellular acidification. In addition, IGF1 altered PySR^−ve/+ve^ phenotypes. These results reveal individual‐specific differences in pyruvate metabolism of normal breast epithelial cells and its association with breast cancer risk.

## 1 INTRODUCTION

Metabolic individuality and its relevance to health risks are currently being explored (Suhre et al., 2011, 2011). Cellular bioenergetics can vary in a given cell type from individual‐to‐individual. This variation can arise due to genetic, epigenetic, aging, and environmental exposures. Bioenergetic impairments are linked with various human diseases including cancer (Wallace, 2005, 2013). Cancer cells reprogram their metabolism to survive, grow, and proliferate. The metabolic reprograming involves changes in bioenergetics and redox balance to support enhanced biosynthesis of macromolecules such as lipids, nucleotides, and nonessential amino acids (DeBerardinis & Chandel, 2016). Alterations in oncogenes and tumor suppressors are considered as the underlying cause of metabolic reprograming in cancer cells. However, it is possible that bioenergetic differences, which in normal cells can be due to genetic variation or environmental exposures, precede cancer development.

Mitochondria play key roles in cellular bioenergetics by carrying out oxidative phosphorylation. Oxidative phosphorylation depends on supply of substrates to the respiratory chain and ATP demand. In most cells, glucose is the primary bioenergetic fuel. After its entry into cells, glucose is converted into pyruvate via the reactions of glycolysis. Next pyruvate is either oxidized inside mitochondria or converted into lactate within cytosol. Pyruvate oxidation inside mitochondria generates NADH and FADH_2,_ which support cellular respiration. The secretion of lactate in the extracellular medium causes acidification, which is often used as a surrogate for assessing the rate of glycolysis (Wu et al., 2007). Because the respiratory chain is functionally linked with the TCA cycle, the bicarbonate produced by it also contributes to medium acidification (Mookerjee, Goncalves, Gerencser, Nicholls, & Brand, 2015). Therefore, relative contributions of lactate and bicarbonate to extracellular medium acidification provide additional insights into overall cellular bioenergetics (Mookerjee, Nicholls, & Brand, 2016).

In this study, we employed respirometry to assess bioenergetic individuality of normal breast epithelial cells from different women and determined if a pre‐existing bioenergetic difference may be linked with breast cancer risk. Further, we assessed bioenergetic responses of breast epithelial cells to treatments with host factors, such as, insulin growth factor 1 (IGF1) and tumor necrosis factor alpha (TNFα) because they were associated with breast cancer risk in women (Kaaks et al., 2014; Szlosarek, Charles, & Balkwill, 2006; To, Knower, & Clyne, 2013). A bioenergetic parameter called pyruvate‐stimulated respiration (PySR) was found to distinguish cells from women with breast cancer and without cancer. The cells from women with breast cancer were mostly PySR^−ve^ (89%). While both cytokines had overall suppressive effects on cellular respiration and acidification, their effects were variable in different individuals. The effects of IGF1 were more consistent compared to TNFα. The PySR^−ve/+ve^ phenotype was also altered by IGF1 to a larger extent than TNFα. Mitochondrial bioenergetics positively regulates tumor suppressor protein p53 and alters radiation sensitivity of cells (Compton et al., 2011). Therefore, we tested the radiation response of HMECs from different individuals in the presence and absence of IGF1. The effects of IGF1 varied in cells from different individuals. These data underscore individual‐specific differences in pyruvate metabolism and they suggest that pre‐existing differences in pyruvate metabolism may contribute to breast cancer risk. The analyses also demonstrate that cytokines can alter breast epithelial cells metabolism.

## 2 MATERIALS AND METHODS

### 2.1 Materials

The reagents were procured from following sources. DMEM/F12 and hydrocortisone were obtained from Corning/Cellgro (Manassas, VA). Mammocult was from StemCell Technologies (Cambridge, MA). Cholera toxin and collagenase were obtained from EMD‐Millipore (Billerica, MA) and Gibco/Thermo Fisher, respectively. Insulin, hyaluronidase, IGF1, TNFα, and all other reagents were purchased from Sigma–Aldrich unless otherwise indicated.

### 2.2 Study outline

**Figure 1:**
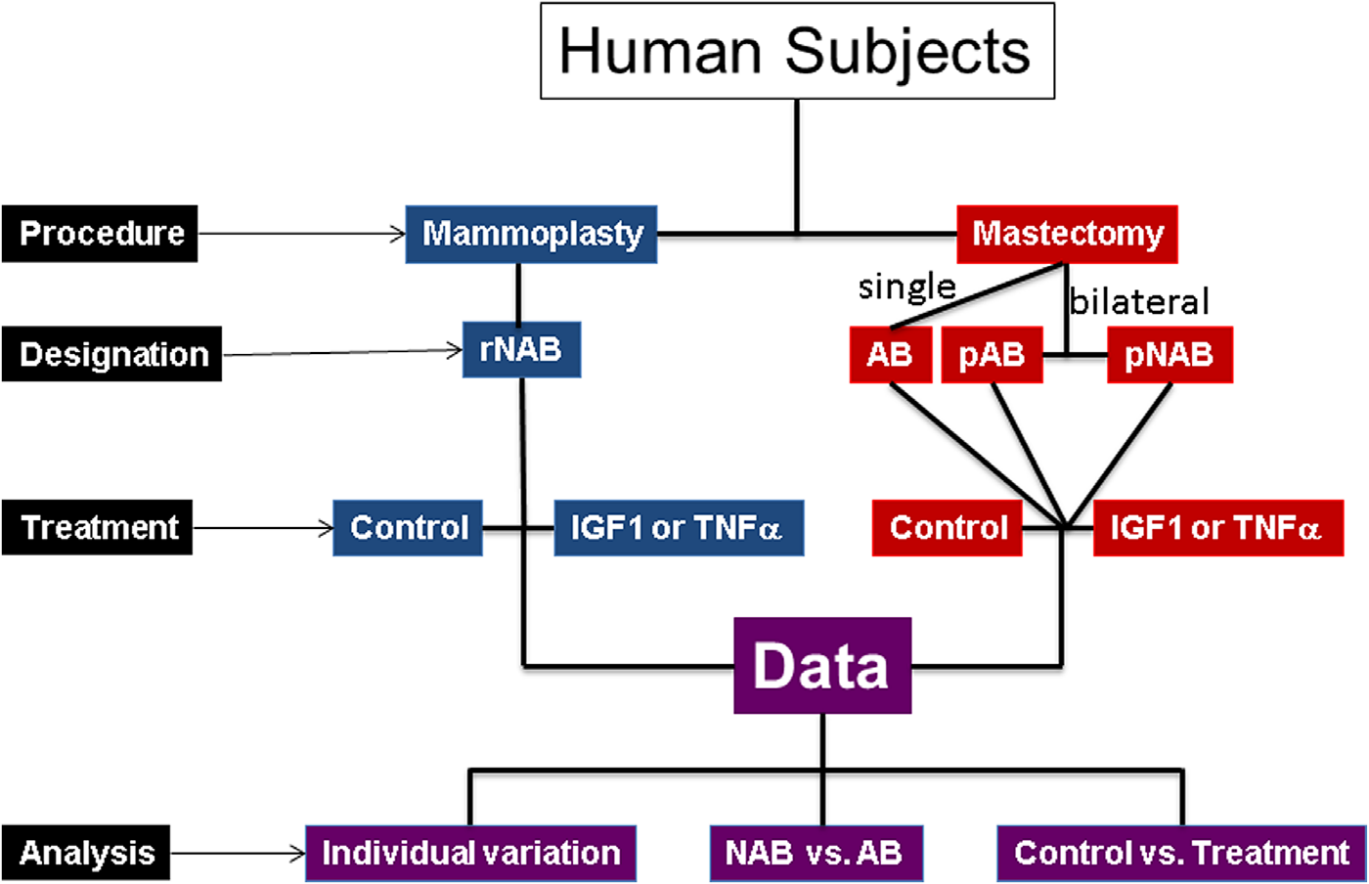
Schematic outline of the study. AB and NAB refer to tumor‐affected or non‐affected breasts, respectively. HMECs designations are: rNAB, reduction mammoplasty‐derived; AB, single mastectomy‐derived; pAB and pNAB, paired bilateral mastectomy‐derived. For more details see Table 1gr1

This study was approved by Baystate Medical Center Institutional Review Board (IRB#324059). The study subjects were women enrolled in the Rays of Hope Breast Research Patient Registry. All subjects were consented to provide excess tissue not needed for diagnostic purposes, and clinical data. Surgery was performed at Baystate Medical Center, Springfield, MA. The procured normal (benign) breast tissue was fresh with less than 1 hr anoxic time. Sampled tissues were from subjects with cancer undergoing unilateral or bilateral mastectomy for cancer, or reduction mammoplasty from subjects with no cancer history (Table 1). In tumor affected breast the normal breast tissue was procured away from the tumor. Normal HMECs obtained from tumor‐affected breasts (AB) and paired non‐affected breasts (NAB) were designated as AB or pAB‐ and pNAB‐HMECs, respectively, (Figure 1). The women undergoing reduction mammoplasty had no history of breast cancer, and HMECs obtained from them were designated rNAB. The pNAB and rNAB were grouped as NAB (unless otherwise specified). In this study only normal/benign HMECs irrespective of their origin in breasts with or without tumors were used. Three types of comparisons were made between AB‐ versus NAB‐HMECs. One involved all AB‐HMECs versus all NAB‐HMECs irrespective of their origin. The second involved pAB versus pNAB‐HMECs obtained from bilateral mastectomies. The third involved pNAB‐ versus rNAB‐HMECs from breast cancer and reduction mammoplasty patients, respectively. Bioenergetics of both AB‐ and NAB‐HMECs with and without IGF1 and TNFα treatments were assessed by in situ respirometry. Respirometry data were analyzed to quantify (i) inter‐individual variation, (ii) bioenergetic differences in AB‐ versus NAB‐HMECs, and (iii) their responses to IGF1 and TNFα treatments. Figure 1 provides a schematic outline of this study.

**Table 1:** List of primary HMECs used in this study

S.N.	HMEC ID	Mammoplasty or mastectomy	Age	BMI	AB‐ HMECs	NAB‐ HMECs
1	110.ROH^a^	Bilateral mastectomy	48	26.7	pAB^a^	pNAB
2	156.ROH	Bilateral mastectomy	61	33.1	pAB	pNAB
3	178.ROH	Bilateral mastectomy	59	27.7	pAB	pNAB
4	179.ROH^a^	Bilateral mastectomy	51	19.1	pAB	pNAB^a^
5	206.ROH	Single mastectomy	46	21.7	AB	
6	207.ROH	Single mastectomy	62	28.1	AB	
7	208.ROH^a^	Single mastectomy	51	26.9	AB^a^	
8	218.ROH	Single mastectomy	49	20.0	AB	
9	219.ROH	Bilateral mastectomy	67	24.7	AB	
10	229.ROH	Bilateral mastectomy	42	22.5	pAB	pNAB
11	231.ROH	Bilateral mastectomy	52	24.1	pAB	pNAB
12	240.ROH	Single mastectomy	71	36.9	AB	
13	245.ROH	Single mastectomy	51	24.6	AB	
14	248.ROH	Bilateral mastectomy	50	26.2	pAB	pNAB
15	250.ROH^a^	Bilateral mastectomy	44	22.3	pAB	pNAB^a^
16	251.ROH	Bilateral mastectomy	49	18.7	pAB	pNAB
17	SS206	Reduction mammoplasty	39	23.6		rNAB
18	SS208	Reduction mammoplasty	43	23.6		rNAB
19	SS212	Reduction mammoplasty	27	22.7		rNAB
20	SS213	Reduction mammoplasty	46	39.0		rNAB
21	SS220	Reduction mammoplasty	36	36.3		rNAB
22	SS229	Reduction mammoplasty	36	23.1		rNAB
23	SS234	Reduction mammoplasty	35	27.5		rNAB
24	SS242	Reduction mammoplasty	46	34.4		rNAB
25	276.ROH^a^	Reduction mammoplasty	19	22.1		rNAB^a^
26	352.ROH^a^	Reduction mammoplasty	56	22.1		rNAB^a^
27	387.ROH^a^	Bilateral mastectomy	27	22.1	AB^a^	

Prefix p in pAB and pNAB indicates paired AB‐ and NAB‐HMECs obtained from the tumor affected (AB) and non‐affected breasts (NAB) of cancer patients. Prefix r in rNAB indicates that NAB‐HMECs came from reduction mammoplasty. HMECs from single mastectomy are denoted as AB as they came from tumor affected breast (AB).

^a^ HMECs used for radiation‐induced death.

### 2.3 HMECs preparation

Tissues were finely minced and digested at 37°C overnight in mammary digestion medium (DMEM/F12 supplemented with 5 µg/ml insulin, 2% BSA, 5 µg/ml hydrocortisone, 10 ng/ml cholera toxin, 300 U/ml collagenase, 100 U/ml hyaluronidase, and 1× antibiotic/antimycotic mixture). Any undigested tissue was removed, and the tissue suspension was centrifuged at 80*g* for 10 min. The pellet was washed in 10 ml of cold Hanks balanced salt solution containing 5% fetal bovine serum (HBF) and re‐centrifuged. Next, the pellet was incubated with 2 ml of 0.25% trypsin/EDTA for 5 min at room temperature, and washed with HBF and centrifuged. The cells were treated with 2 ml dispase (2 mg/ml) and 20 U of DNase‐I for 5 min at room temperature before HBF wash and centrifugation. Cells were passed through 100 and 40 μm cell strainers and centrifuged for 5 min at 100*g*. Resulting cells were designated as HMECs, plated in 10% fetal bovine serum to allow adherence and then switched to Mammocult medium for culture with designation Passage zero (P0). Subsequently, HMECs were passaged at 3–4 day intervals, and all experiments were carried out with HMECs at passage two unless otherwise noted. Table 1 shows the list of HMECs used in this study with associated age and body mass index (BMI) of the subjects.

### 2.4 In situ respirometry and extracellular acidification analysis

**Figure 2:**
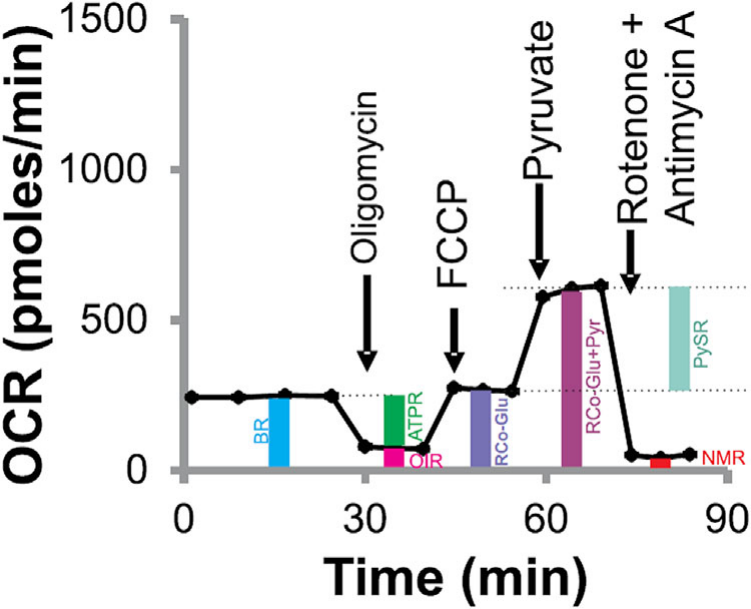
A representative respiratory profile. It shows the experimental scheme, pyruvate‐stimulated respiration (PySR) and selected bioenergetic parameters. Indicated compounds were added sequentially from left to right. See Table 2 for individual bioenergetic parameters, and corresponding abbreviations, that can be derived from such a respiratory profile. Averages of at least three oxygen consumption rates (OCR) for each parameter were used in different analyses. On *x*‐axis each dot represents one rate at corresponding time

Oxygen consumption rates (OCR) were measured using a XF24‐3 Analyzer (Agilent‐Seahorse, Billerica, MA). Cells were cultured in V7 PS plates (10,000–80,000/well) in complete Mammocult medium containing 5% fetal bovine serum without antibiotics for ∼48 hr at 37°C in a 5% CO_2_ incubator before respiration measurements. Antibiotics‐free medium was used for 48 hr culture to remove any suppressive effects of antibiotics on mitochondrial protein synthesis. The IGF1 (20 ng/ml) and TNFα (10 ng/ml) were added to cells 24 hr before respiration assays and 24 hr post‐seeding. The respiratory activity of cells was assayed in 700 µl low‐K^+^ buffer [LKB: 3.5 mM KCl, 10 mM KH_2_PO_4_, 1 mM Na_2_SO_4_, 2 mM MgCl_2_, 1.3 mM CaCl_2_, 120 mM NaCl, 15 mM glucose, pH 7.4] containing 0.4% fatty acids‐free bovine serum albumin (BSA). Cells were washed twice with 700 µl LKB and then incubated in a non‐CO_2_ incubator at 37°C for ∼30–60 min. Pre‐hydrated XF24 cartridges for 24 hr were calibrated according to the manufacturer's (Agilent‐Seahorse) instructions after loading injection ports with the indicated compounds (75 µl). After calibration of the sensor cartridges, the V7 culture plates with cells were loaded into the XF24‐3 analyzer. Repeated cycles of mixing, waiting, and measurements were performed as described (Gerencser et al., 2009; Wu et al., 2007). After four basal respiration rate measurements, oligomycin (2 µg/ml), carbonylcyanide p‐trifluoromethoxy phenylhydrazone (FCCP, 2 µM), pyruvate (10 mM), and rotenone plus antimycin A (1 µM each) were added. At least three respiration rates were measured following the addition of each compound (Figure 2). The obtained respiratory profiles were used to derive different parameters as in Table 2. Means of four basal rates and three rates after treatments (*n* = 4–5 well/group) were used for data analysis (see below).

**Table 2:** Bioenergetic parameters derived from a respiratory profile as shown in Figure 2

Parameters descriptions—>	Abbreviation
Basal respiration (see Figure 2)	BR
Non‐mitochondrial respiration (rotenone + antimycin‐A insensitive; see Figure 2)	NMR
Mitochondrial basal respiration (BR‐NMR)	mBR
Oligomycin‐insensitive respiration (see Figure 2)	OIR
ATP synthesis supporting respiration (oligomycin‐sensitive, BR‐OIR)	ATPR
Proton‐leak supporting respiration (OIR‐NMR)	PLR
Respiratory capacity (FCCP‐induced respiration, o = oligomycin present)	RCo
Mitochondrial respiratory capacity (RCo‐NMR)	mRCo
Pyruvate‐stimulated respiration	PySR
Coupling efficiency (ATPR/mBR)	CE
Mitochondrial respiratory control ratio (mRC_O_/ATPR)	RCR
Mitochondrial spare respiratory capacity with oligomycin (RCo‐BR/mRCo)	SRC
Apparent respiratory state (4‐[ATPR/mRCo‐OIR])	State

Units for respirations concerning BR, NMR, mBR OIR, ATPR, PLR, RCo, mRCo, and PySR are pmoles O_2_/min or pmoles O_2_/min/µg protein. Units for CE, RCR, and SRC are ratio, which can be converted to % by multiplying by 100. Apparent respiratory State is unitless.

Pilot experiments were performed to optimize seeding cell density and compare OCR and extracellular acidification rates (ECAR) of HMECs from the same subject at P2 and P4. Basal OCR and ECAR values of P2 HMECs showed linear response with cell density (OCR, *R* ^2^ = 0.89; ECAR, *R* ^2^ = 0.95, data not shown). Because in vivo HMECs are in contact with each other, we chose higher cell density (80,000/well) within linear range for all experiments. P2 cells gave better OCR and ECAR values than P4 cells (not shown). Thus assays were performed with P2 HMECs. The protein content in each well was assayed at the end of the experiment by adding 120 µl/well of RIPA lysis buffer (50 mM Tris.HCl, 150 mM NaCl, 100 mM NaF, 10 mM MgCl_2_, 0.5% NP40) containing protease inhibitors cocktail #P1860 (1:100 dilution, Sigma–Aldrich). A 25 µl aliquot of lysate from each well was used to measure protein concentration by Pierce BCA protein assay kit (Prod# 23225; Thermo Scientific, Rockford, IL). Protein concentration was calculated using BSA standard curves. The respirometry data were corrected for equal protein content for comparisons unless otherwise noted.

The extracellular acidification rates (ECAR) obtained from the XF24‐3 Analyzer were used to calculate total, respiratory, and glycolytic proton production rates (PPRtotal, PPRresp, and PPRglyc, respectively) as described (Mookerjee et al., 2015). Buffering power (0.0322) of the respiratory medium was used to determine the fractions of PPRresp and PPRglyc as described (Mookerjee & Brand, 2015). OCR and ECAR values related to basal and treated conditions (oligomycin, FCCP, pyruvate, rotenone + antimycin A) were used to calculate PPRresp and PPRglyc under each condition. Unless otherwise indicated PPRtotal, ‐resp, and ‐glyc values for basal and oligomycin‐treated conditions are reported.

### 2.5 Metabolic phenotyping/fingerprinting

Metabolic phenotyping was carried out using 96‐well PM‐M1 phenotyping microarray for mammalian cells (BioLog Inc., Hayward, CA; (Bochner et al., 2011)). PM‐M1 microarray was preloaded with different carbon substrates. Cells (10,000/well) were seeded in 50 µl IF‐M1 medium supplemented with 5% dialyzed serum in PM‐M1 plates. After 24 hr incubation at 37°C in a 5% CO_2_ incubator, 10 µl redox dye MA was added. Plates containing control and IGF1‐treated cells were processed in parallel. Absorbances at 590 and 750 nm wavelengths were recorded at different time points and absolute A_590–750_ and OD/min within 30–60 min after MA dye addition were compared for scoring the difference between control and IGF1‐treated cells.

### 2.6 Radiation sensitivity of HMECs

HMECs were plated in 8‐well glass chamber slides (Falcon, BD Biosciences) and allowed to settle overnight. Half the cultures were pretreated with 20 ng/ml IGF‐1 for 1 hr and then they were exposed to 5 Gy of γ‐radiation using a ^137^Cs irradiator (Gammator‐B, Radiation Machinery Corporation, Parsippany, NJ). Next 24 hr after irradiation, the cells were checked for viability using the Live/Dead assay kit (Invitrogen). Briefly, 2 μM for Calcein‐AM and 4 μM for EthD‐1 (Invitrogen/Thermo Fisher) were added to the cells in PBS and incubated at 37°C for 3 hr. Then cells were imaged by fluorescent microscopy. Image J was used for counting of live and dead cells. The percentage of cell death in control and IGF1‐treated conditions was compared using GraphPad Prism 5 software.

### 2.7 Data analysis

AB‐ versus NAB‐, pAB‐ versus pNAB‐, and pNAB‐ versus rNAB‐HMECs comparisons were made using unpaired Student's *t*‐test. Corresponding controls versus treated (IGF1 or TNFα) comparisons were made using paired Student's *t*‐test. Averages of four basal rates and three rates of treated conditions after the addition of indicated compound(s) were used in analyses. Statistical analyses were performed mostly using GraphPad Prism 5 and Microsoft Excel. Data are shown either as mean ± SD or mean ± sem as specified. Means of each bioenergetic parameter between two groups were compared by Student's *t*‐test as indicated above. Statistical significance was calculated at p* ≤ 0.05, **0.01, ***0.001 with 95% confidence interval. The frequency of PySR^−ve^ or PySR^+ve^ phenotypes in pNAB‐ versus rNAB‐HMECs samples was analyzed by Chi‐square test using GraphPad Prism 5. The 20% cut‐off for PySR^+ve^ phenotype was arbitrarily set above the average standard error of controls, IGF1‐ and TNFα‐treated rNAB‐HMECs, because mostly they showed the PySR^+ve^ phenotype and they were from women who did not have cancer.

## 3 RESULTS

### 3.1 Inter‐individual variation in AB‐ and NAB‐HMECs bioenergetics

The variation in bioenergetics of HMECs from different women was assessed by respirometry. Figure 2 shows a typical respiratory profile with sequential additions of oligomycin, FCCP, pyruvate, and rotenone plus antimycin. Such profiles were obtained for HMECs from different individuals and used to derive various bioenergetic parameters as indicated in Table 2. Briefly, basal respiration has three components: ATP synthesis (ATPR)—and H^+^‐leak (PLR)—supporting respirations, and non‐mitochondrial respiration (NMR) (Jekabsons & Nicholls, 2004). The respiration medium, LKB, contained 15 mM glucose from the start. Thus basal respiration was primarily supported by glucose and any internal substrates that might be present inside cells. The oligomycin was added to block ATP synthesis and to estimate ATPR. The oligomycin‐insensitive respiration after subtracting the NMR gave an estimate of PLR. FCCP was used to induce maximal respiration by dissipating the H^+^ gradient across the mitochondrial inner membrane. Thus FCCP addition after oligomycin reports respiratory capacity on glucose (RCo). Blocking mitochondrial synthesis may limit respiratory capacity due to ATP limitation. Therefore, to overcome any limitation in respiratory capacity, 10 mM pyruvate was added after FCCP. By using this experimental scheme, as shown in Figure 2, it was possible to assess respiratory capacity of cells on glucose versus glucose + pyruvate as substrates (i.e., before and after pyruvate addition, respectively) within the same experiment and assess individual‐specific responses to exogenous pyruvate.

**Figure 3:**
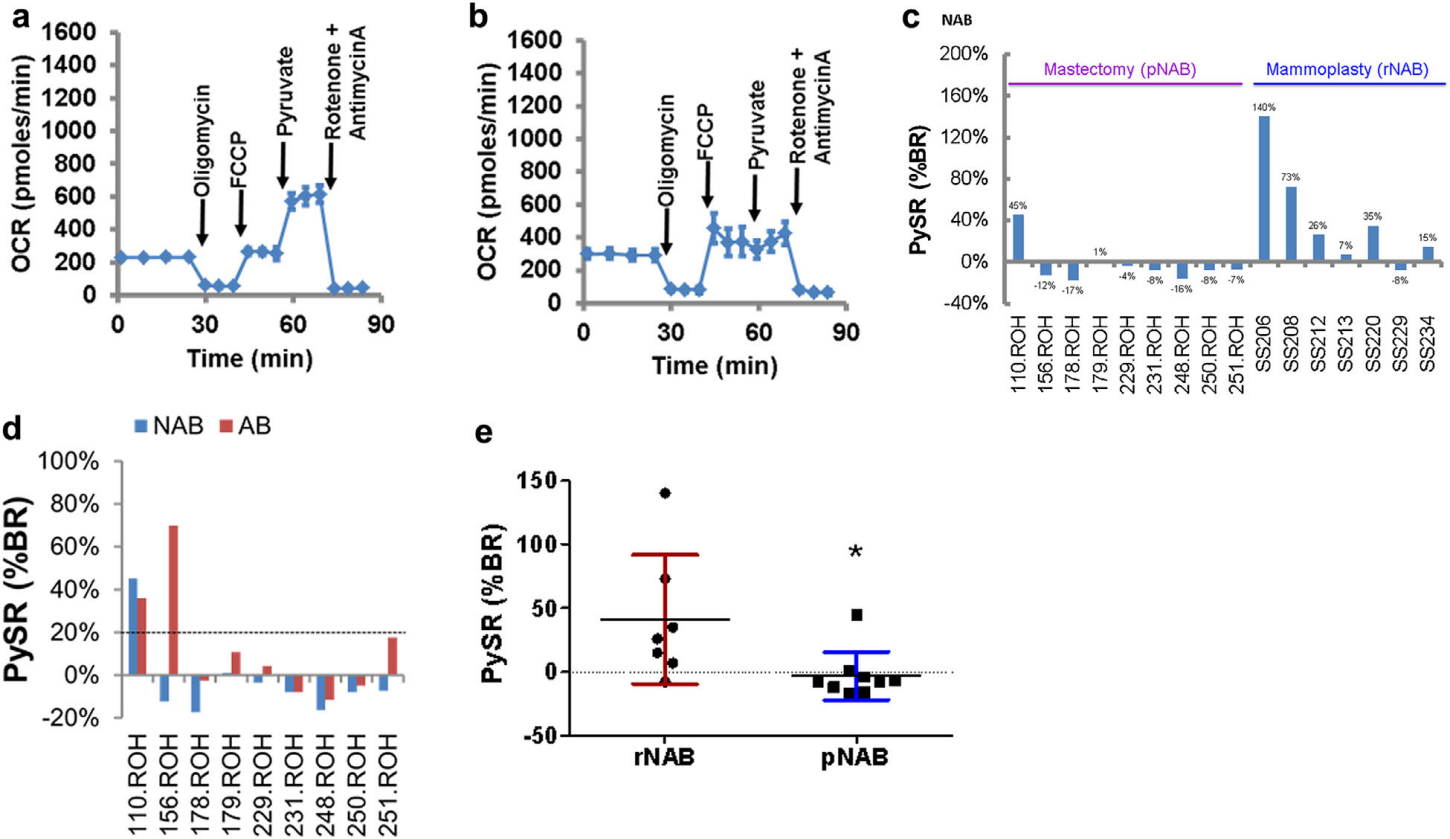
Individual‐specific variation in the respiratory activity. a) A respiratory profile of rNAB‐HMECs (SS206) from a woman displaying robust response to exogenous pyruvate. b) A respiratory profile of rNAB‐HMECs (SS229) from a woman displaying no response to exogenous pyruvate. c) Inter‐individual variation in PySR in pNAB‐ versus rNAB‐HMECs. The pNAB‐ and rNAB‐HMECs were derived from bilateral (prophylactic) mastectomy (*n* = 9) and reduction mammoplasty (*n* = 7), respectively. See Figure 1 and Table 1 for details. Samples showing PySR ≥ 20% of basal respiration rate (BR) were designated PySR^+ve^ and the rests were considered as PySR^−ve^. Average NAB control values from Tables 5 and 6 are plotted here. d) Minimal PySR^−ve/+ve^ phenotype switching between corresponding pAB‐ and pNAB‐HMECs. Broken line marks the 20% cut‐off. e) Relative PySR in rNAB‐ (*n* = 7) versus pNAB‐HMECs (*n* = 9) (mean ± SD, unpaired *t*‐test *p* = 0.029)

Pyruvate metabolism plays a central role in cellular bioenergetics. Therefore, we compared respiratory responses of HMECs from different women to exogenous pyruvate. Figure 3a and b show the effects of pyruvate on respiratory profiles of rNAB‐HMECs from two women (SS206 and SS229). While cells from SS206 showed pyruvate‐stimulated respiration (PySR, Figure 3a), the cells from SS209 did not (Figure 3b). Pyruvate increased respiration by 123% in SS206‐HMECs and decreased by 6% in SS29‐HMECs. This suggests that respiratory response to exogenous pyruvate varies in breast epithelial cells from different individuals. To assess the variation in respiratory response to pyruvate, we classified samples as PySR^−ve^ and PySR^+ve^. The difference in FCCP‐stimulated respiration before and after pyruvate addition was divided with basal respiration to define the degree of variation in PySR (%BR). Since basal respiration is not subjected to change by treatments, the normalization with basal respiration gives an estimate of the degree of PySR variance across samples. Samples showing PySR by ≥20% of basal respiration were considered PySR^+ve^ and the rest were considered as PySR^−ve^. By this criterion 31% NAB‐HMECs (*n* = 5/16) were PySR^+ve^ (Figure 3c). These data suggest that in the majority of NAB‐HMECs, glucose was sufficient to support maximal respiration and exogenous pyruvate did not increase respiration any further. Therefore, pyruvate addition did not alter mean spare respiratory capacity (SRC) significantly in either AB‐ or NAB‐HMECs (Table 3). However, there were individual‐specific differences. In some samples, SRC values were negative due to a decline of respiration following FCCP addition. In these samples, the addition of pyruvate did not rescue the respiratory capacity. This could be either due to defective respiratory chain, redox homeostasis, or pyruvate delivery to mitochondria. These data support existence of individual‐ specific variation in pyruvate metabolism of breast epithelial cells.

**Table 3:** Effect of pyruvate on spare respiratory capacity before (SRCglu) and after pyruvate addition (SRCglu + pyr)

AB			NAB		
ID	SRCglu	SRCglu + pyr	ID	SRCglu	SRCglu + pyr
110.ROH	42%	45%	110.ROH	59%	64%
156.ROH	66%	75%	156.ROH	32%	30%
178.ROH	5%	6%	178.ROH	34%	24%
179.ROH	21%	31%	179.ROH	52%	57%
206.ROH	51%	55%	229.ROH	28%	24%
207.ROH	37%	41%	231.ROH	4%	3%
208.ROH	33%	30%	248.ROH	−4%	−31%
218.ROH	−12%	−11%	250.ROH	−87%	−110%
219.ROH	20%	16%	251.ROH	21%	19%
229.ROH	−21%	−10%	SS206	10%	64%
231.ROH	−92%	−113%	SS208	34%	56%
240.ROH	34%	32%	SS212	60%	65%
245.ROH	−52%	−73%	SS213	−72%	−50%
248.ROH	−8%	−22%	SS220	27%	45%
250.ROH	−72%	−75%	SS229	24%	18%
251.ROH	22%	37%	SS234	28%	37%
n	16	16	n	16	16
Mean	5%	4%	Mean	16%	20%
SD	45%	52%	SD	41%	48%
SE	11%	13%	SE	10%	12%
*p*‐value	0.816		*p*‐value	0.410	

Means were compared by paired Student's *t*‐test and SRCglu versus SRCglu + pyr *p* values are given.

To determine whether the PySR^−ve^ or PySR^+ve^ phenotype was more common in breast epithelial cells from women without cancer, we compared pNAB‐ versus rNAB‐HMECs. There was a striking difference in PySR^−ve^ versus PySR^+ve^ frequencies of both groups (Figure 3c). The majority of pNAB‐HMECs were PySR^−ve^. On the other hand, the majority of rNAB‐HMECs (57%; *n* = 4/7) were PySR^+ve^. The average age of women undergoing mammoplasty was significantly lower than the women undergoing mastectomy (37.43 ± 6.13 vs. 50.57 ± 6.20 years, respectively, mean ± SD, unpaired *t*‐test, *p* = 0.001). However, there was no correlation between PySR^+ve^ phenotype and age (Pearson *r* = −0.3988, *p* = 0.1260). Also, there was no significant difference in body mass index (BMI) between pNAB versus rNAB samples (27.97 ± 6.84 vs. 25.43 ± 4.03, mean ± SD, unpaired *t*‐test, *p* = 0.40). The mean PySR between pNAB‐ versus rNAB‐HMECs differed significantly (−3.03% ± 19.02% vs. 41.15% ± 50.50%, mean ± SD, *n* = 7 vs. 9, *t*‐test *p* = 0.029; Figure 3e). These data suggest that PySR^−ve^ phenotype may be associated with breast cancer risk. This is supported by predominantly PySR^−ve^ phenotype of AB‐HMECs (*n* = 14/16).

To determine the degree of PySR^−ve/+ve^ phenotype switching in cells from the same women, we compared pAB‐ versus pNAB‐HMECs. The pAB‐ and pNAB‐HMECs were obtained from bilateral mastectomy. We noted about 33% (3/9) pAB‐HMECs differed from the corresponding pNAB‐HMECs (Figure 3d). Based on 20% cut‐off, only 1/9 sample became PySR^+ve^ from PySR^−ve^ (11%; *n* = 1/9; Figure 3d, 156.ROH). Thus, there was minimal PySR^−ve/+ve^ phenotype switching between pAB‐ versus pNAB‐HMECs (*p* = 0.527, Chi‐square test). Together, these data suggest that there is a good agreement in PySR^−ve^ phenotype of breast epithelial cells from cancer patients, whether they come from the tumor affected or non‐affected breasts. These data also reveal individual‐specific bilateral differences in local environments due to the presence of tumor.

**Figure 4:**
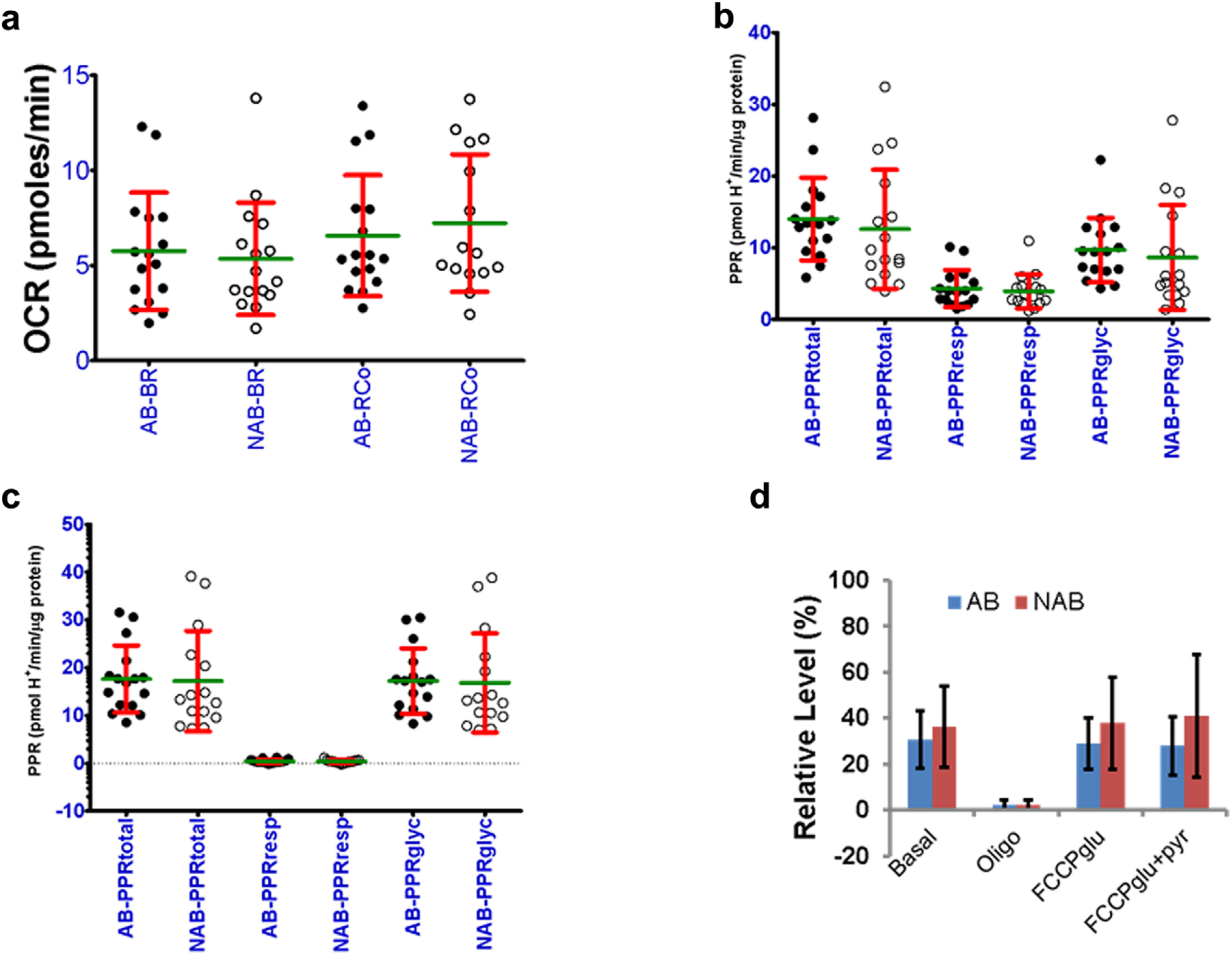
Individual‐specific variation in respiration and extracellular acidification rates. a) Variations in basal (BR) and maximal (RCo) respiratory activities of AB‐ versus NAB‐HMECs. Protein normalized values (OCR:pmoles/min/µg protein, mean ± SD) are shown. BR and RCo in comparisons of AB versus NAB were not significantly different at *p* ≤ 0.05 (unpaired Student's *t*‐test). b) Variation in proton production rate (PPR) by AB‐ versus NAB‐HMECs under basal condition. PPRtotal, ‐resp, and ‐glyc refer to total, respiratory and glycolytic PPRs. c) Variation in PPR by AB‐ versus NAB‐HMECs following oligomycin treatment. d) Percent contribution of PPRresp to PPRtotal under different conditions

Next, we determined fold variation (max/min) in respiration and extracellular acidification rates of HMECs from different women. Both AB‐ and NAB‐HMECs showed at least sixfold variation in basal respiration and about fivefold variation in maximal respiration (Figure 4a; BR, RCo). The acidification of respiratory medium by cells is a measure of lactate and bicarbonate secretions. The lactate‐ and bicarbonate‐mediated acidifications inform about relative contributions of glycolysis (PPRglyc) and respiration (PPRresp) (Mookerjee et al., 2015). We calculated the proton production rates (PPR) by AB‐ and NAB‐HMECs under different conditions. We noted about six‐ to eight‐fold variation in basal PPR and 5‐ to 16‐fold variation in oligomycin‐induced PPR in both AB‐ and NAB‐HMECs from different women (Figure 4b,c). Under basal conditions 30–36% acidification was due to respiration‐associated bicarbonate secretion (Figure 4d). In oligomycin‐treated cells, the respiratory acidification declined to ∼2% in both AB‐ and NAB‐HMECs. This is expected because the TCA cycle will be slowed down in the presence of oligomycin (Kim et al., 2014). Addition of FCCP after oligomycin increased the respiratory acidification to 29–39% of total. This is expected because the TCA cycle activity will increase to supply NADH/FADH_2_ to the respiratory chain. Addition of pyruvate after FCCP did not increase either respiratory or glycolytic acidification any further (Figure 4d, data not shown). Together, the above data demonstrate the existence of a considerable degree of variation in respiration and acidification rates of normal breast epithelial cells from different women.

The coupling efficiency (CE) informs about the degree of basal respiration supporting ATP synthesis inside mitochondria. Therefore, we assessed variation in coupling efficiency, which ranged from 85% to 105% in AB‐HMECs and 84% to 110% in NAB‐HMECs. Its values above 100% are due to over‐estimation of NMR. Overall the NMR was 21 ± 3% and 23 ± 3% of basal respiration in AB‐ and NAB‐HMECs (mean ± sem, *n* = 16 each group, Table 4). These values were obtained in the presence of oligomycin and FCCP added as shown in Figure 2. However, without any prior treatments with oligomycin and FCCP, the NMR values were comparable (20 ± 1%, mean ± sem, NAB‐HMECs, *n* = 5, not shown). Thus ∼80% of basal respiratory activity in normal breast epithelial cells supports respiratory chain function. Of this 7 ± 2% and 6 ± 1% supports H^+^ leak in AB‐ and NAB‐HMECs, respectively. Together, the above data suggest that there is considerable variation in the respiratory activity, pyruvate metabolism, spare respiratory capacity, coupling efficiency, and extracellular acidification rate of breast epithelial cells from different women. Despite considerable variation about 80% of basal respiration supports respiratory chain function with the majority contributing to ATP synthesis.

**Table 4:** Respiration rates supporting different bioenergetic parameters in AB‐ versus NAB‐ HMECs

	AB				NAB			
Parameter	*N*	Mean	SD	SEM	*N*	Mean	SD	SEM
BR	16	5.76	3.07	0.77	16	5.35	2.96	0.74
NMR	16	1.23	0.58	0.15	16	1.22	0.58	0.14
mBR	16	4.53	2.71	0.68	16	4.14	2.50	0.62
OIR	16	1.65	0.81	0.20	15	1.61	0.82	0.21
ATPR	16	4.18	2.55	0.64	15	3.86	2.25	0.58
PLR	16	0.42	0.41	0.10	15	0.37	0.35	0.09
RCo	16	6.57	3.18	0.80	16	7.32	3.51	0.88
mRCo	16	5.34	2.69	0.67	16	6.09	3.18	0.79
PySR	16	0.22	0.87	0.22	16	0.72	1.86	0.47
CE	16	0.92	0.07	0.02	15	0.93	0.07	0.02
RCR	16	1.53	0.84	0.21	15	1.70	0.75	0.19
SRC	16	0.08	0.47	0.12	16	0.25	0.42	0.11
State	16	3.06	0.54	0.13	15	3.20	0.50	0.13

Maximal respiratory capacities (RCo, mRCo) are reported here irrespective of whether they were obtained on glucose alone or glucose + pyruvate as substrates. Units for respiration concerning BR, NMR, mBR OIR, ATPR, PLR, RCo, mRCo, and PySR are pmoles O_2_/min/µg protein. Units for CE, RCR, and SRC are ratio that can be converted to % by multiplying with 100. Apparent respiratory State is unitless. One sample did not show a clear effect of oligomycin in the NAB group. Therefore it is excluded from the analysis of parameters affected by oligomycin. None of the reported parameters differ significantly between AB versus NAB group (unpaired Student's *t*‐test at *p* = 0.05).

The presence of tumor may alter the bioenergetics in surrounding normal epithelial cells. If the differences are stable, then they can be revealed by comparing AB‐ and NAB‐HMECs cells. Therefore, we compared the means of different parameters between AB‐ and NAB‐HMECs. Our analyses did not reveal a significant difference in any of the parameters including basal and maximal respiration rates (BR, RCo), CE, ATPR, PLR, SRC, and apparent respiratory state (Table 4, Figure 4a). The PPRtotal, PPRresp, and PPRglyc were also comparable (Figure 4b,c). Like AB‐ versus NAB‐HMECs, no significant difference between pAB‐ versus pNAB‐HMECs was observed (not shown). Between pNAB‐ and rNAB‐HMECs, the only notable difference was the effect of exogenous pyruvate, which is discussed above. Although, the glycolytic PPR in rNAB‐HMECs was relatively lower than pNAB‐HMECs, it was not statistically significant (4.98 ± 7.21 rNAB *n* = 7 vs. 11.94 ± 8.29 pNAB *n* = 9, mean ± SD). Therefore, we conclude that overall mitochondrial bioenergetics of benign epithelial cells from cancer affected and non‐affected breasts are similar despite individual‐specific variation.

### 3.2 Bioenergetic response of HMECs to IGF1 treatment

**Figure 5:**
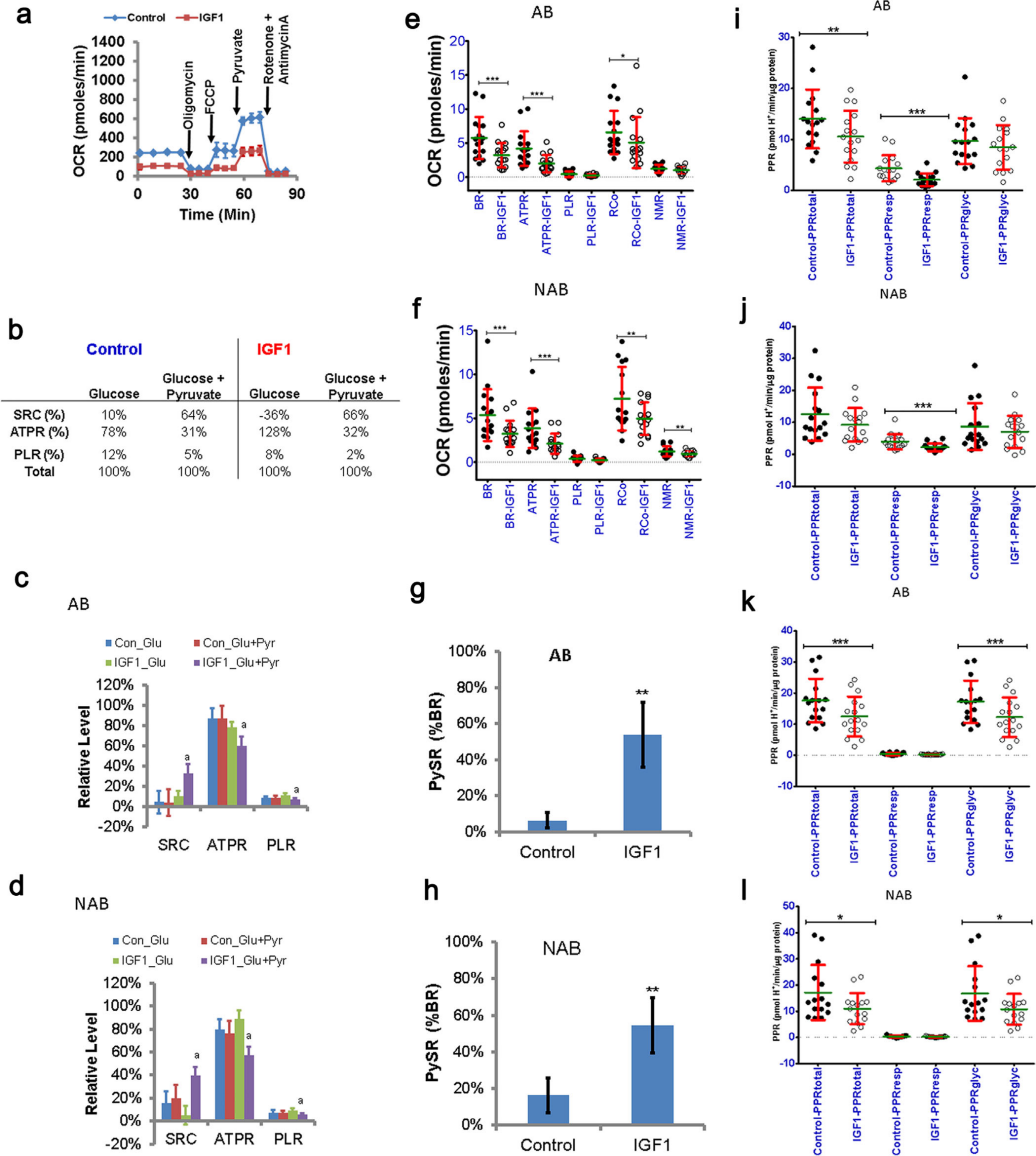
Bioenergetic response of HMECs to IGF1 treatment. a) Respiratory profiles of control versus IGF1‐treated SS206 HMECs. b) Effect of IGF1 on mitochondrial bioenergetics of SS206 HMECs. Values calculated based on the respiratory profiles in panel A. c) Effect of IGF1 on mitochondrial bioenergetics of AB‐HMECs (*n* = 16, mean ± sem, ^a^IGF1_Glu versus IGF1_Glu + Pyr **p ≤ 0.01). d) Effect of IGF1 on mitochondrial bioenergetics of NAB‐HMECs (*n* = 16, mean ± sem, ^a^IGF1_Glu vs. IGF1_Glu + Pyr, **p ≤ 0.01 [SRC, ATPR], * ≤ 0.05 [PLR]). e) Effect of IGF1 on respiration rates supporting different bioenergetic parameters in AB‐HMECs (*n* = 16, mean ± SD, OCR:pmoles/min/µg protein, *p ≤ 0.05, *** ≤ 0.001). f) Effect of IGF1 on respiration rates supporting different bioenergetic parameters in NAB‐HMECs (*n* = 16, mean ± SD, OCR/pmoles/µg protein, **p ≤ 0.01, *** ≤ 0.001). g) Effect of IGF1 on PySR in AB‐HMECs (*n* = 16, mean ± sem, **p ≤ 0.01). h) Effect of IGF1 on PySR in NAB‐HMECs (*n* = 16, mean ± sem, **p ≤ 0.01). i) Effect of IGF1 on basal PPR in AB‐HMECs (*n* = 16, mean ± SD, Control vs. IGF1 **p ≤ 0.01, *** ≤ 0.001). j) Effect of IGF1 on basal PPR in NAB‐HMECs (*n* = 16, mean ± SD, Control vs. IGF1, *** ≤ 0.001). k) Effect of IGF1 on PPR in AB‐HMECs after oligomycin addition (*n* = 16, mean ± SD, Control vs. IGF1 ***p ≤ 0.001). l) Effect of IGF1 on PPR in NAB‐HMECs after oligomycin addition (*n* = 16, mean ± SD, Control vs. IGF1 *p ≤ 0.05)

We tested IGF1 as a potential host‐factor that could affect cellular bioenergetics, because IGF1 is known to be associated with breast cancer risk (Kaaks et al., 2014). Cells were treated with IGF1 for 24 hr before respiratory assays were performed. Respirometry profiles of control and IGF1‐treated cells were obtained in side‐by‐side assays and different bioenergetic parameters were calculated. Figure 5a shows an example of reduced respiratory activity in IGF1‐treated cells from one individual (SS206). The corresponding parameters indicative of mitochondrial bioenergetics are shown in Figure 5b. Clearly in IGF1‐treated SS206 cells SRC was reduced below basal level (−36%), which was rescued by pyruvate addition. This suggests that in these cells glucose metabolism was inadequate to support maximal respiration. Because PySR^+ve^ phenotype was less frequent than PySR^−ve^ phenotype, we did not observe a significant difference in SRC, ATPR, and PLR values between glucose alone and glucose+ pyruvate in control AB‐ and NAB‐HMECs (Figure 5c,d; Con_Glu vs. Con_Glu + Pyr). However, in the IGF1 treated cells a significant difference between glucose alone and glucose + pyruvate conditions was observed in both AB‐ and NAB‐HMECs (Figure 5c,d: IGF1_Glu vs. IGF1_Glu + Pyr). SRC was higher in IGF1‐treated cells on glucose + pyruvate together (AB: 5 ± 11% vs. 33 ± 9%, *p* = 0.001, NAB 16 ± 10% vs. 39 ± 8%, *p* = 0.002; mean ± sem, *n* = 16 each group). The difference in SRC was primarily a consequence of reduced basal respiration (Figure 5e). In addition to basal respiration, ATPR and RCo were also significantly lower in IGF1 treated cells (Figure 5e,f). Overall, IGF1 had suppressive effects on respiration of both AB‐ and NAB‐HMECs.

PySR informs about differences in exogenous pyruvate oxidation. Therefore, we determined how IGF1 treatment affected PySR^+ve/−ve^ phenotype. We observed an increase in the fraction of samples showing PySR^+ve^ phenotype in both AB‐ and NAB‐HMECs following treatment with IGF1. In AB‐HMECs, the PySR^+ve^ fraction increased from 13% (*n* = 2/16) to 63% (*n* = 10/16; Table 5). In NAB‐HMECs, it increased from 31% (*n* = 5/16) to 60% (*n* = 11/16; Table 5). The increase in PySR^+ve^ fraction affected the mean PySR values significantly in both AB‐ and NAB categories (Figure 5g,h). These and above mentioned data suggest that: (i) IGF1 can suppress respiratory activity, (ii) alter respiratory response to exogenous pyruvate, and (iii) affect mitochondrial bioenergetics of epithelial cells from both tumor‐affected and non‐affected breasts.

**Table 5:** Effect of IGF1 on PySR

AB			NAB		
ID	Control	IGF1	ID	Control	IGF1
110.ROH	5%	100%	110.ROH	20%	169%
156.ROH	61%	255%	156.ROH	−4%	2%
178.ROH	2%	−3%	178.ROH	−16%	−12%
179.ROH	13%	85%	179.ROH	17%	85%
206.ROH	14%	80%	229.ROH	−4%	71%
207.ROH	7%	40%	231.ROH	−2%	22%
208.ROH	−5%	22%	248.ROH	−15%	−15%
218.ROH	1%	4%	250.ROH	−4%	2%
219.ROH	−4%	23%	251.ROH	−3%	25%
229.ROH	6%	49%	SS206	135%	172%
231.ROH	−5%	−5%	SS208	61%	31%
240.ROH	−4%	156%	SS212	26%	76%
245.ROH	−7%	−1%	SS213	7%	5%
248.ROH	−9%	1%	SS220	35%	120%
250.ROH	−1%	−12%	SS229	−8%	24%
251.ROH	24%	69%	SS234	15%	94%
*n*	16	16	*n*	16	16
Mean	6%	54%	Mean	16%	54%
SD	17%	71%	SD	38%	61%
SE	4%	18%	SE	9%	15%
*p*‐value	0.006		*p*‐value	0.004	

Means of control versus IGF1‐treated cells were compared by paired Student's *t*‐test and *p* values are given.

To determine if the suppressive effect of IGF1 on respiration correlated with reduced extracellular acidification, we compared the proton production rates (PPR) in control versus IGF1‐treated cells. Under basal condition, IGF1 significantly reduced respiratory PPR in both AB‐ and NAB‐HMECs (Figure 5i,j). This correlated with a significant reduction in total PPR in AB‐HMECs only. However in oligomycin treated condition, IGF1 significantly reduced glycolytic PPR that correlated with reduction in total PPR in both AB‐ and NAB‐HMECs (Figure 5k,l). This suggests that mitochondrial ATP synthesis supports glycolysis in IGF1‐treated cells to a larger extent than control cells. Under FCCP‐treated conditions, both respiratory and glycolytic acidifications contributed toward reduced total acidification (Table S1). Unlike in AB‐HMECs, in the presence of exogenous pyruvate, glycolytic PPR was not significantly affected by IGF1 in NAB‐HMECs (Table S1). In terms of percent contribution of respiratory and glycolytic PPRs, the AB‐HMECs were different from the NAB‐HMECs (Table S2). These data suggest that IGF1 suppresses respiratory activity of HMECs by suppressing glycolysis. Further, in terms of extracellular acidification, there is a potential difference in the metabolism of breast epithelial cells from tumor‐affected and non‐affected breasts in response to IGF1.

### 3.3 Bioenergetic response of HMECs to TNFα treatment

**Figure 6:**
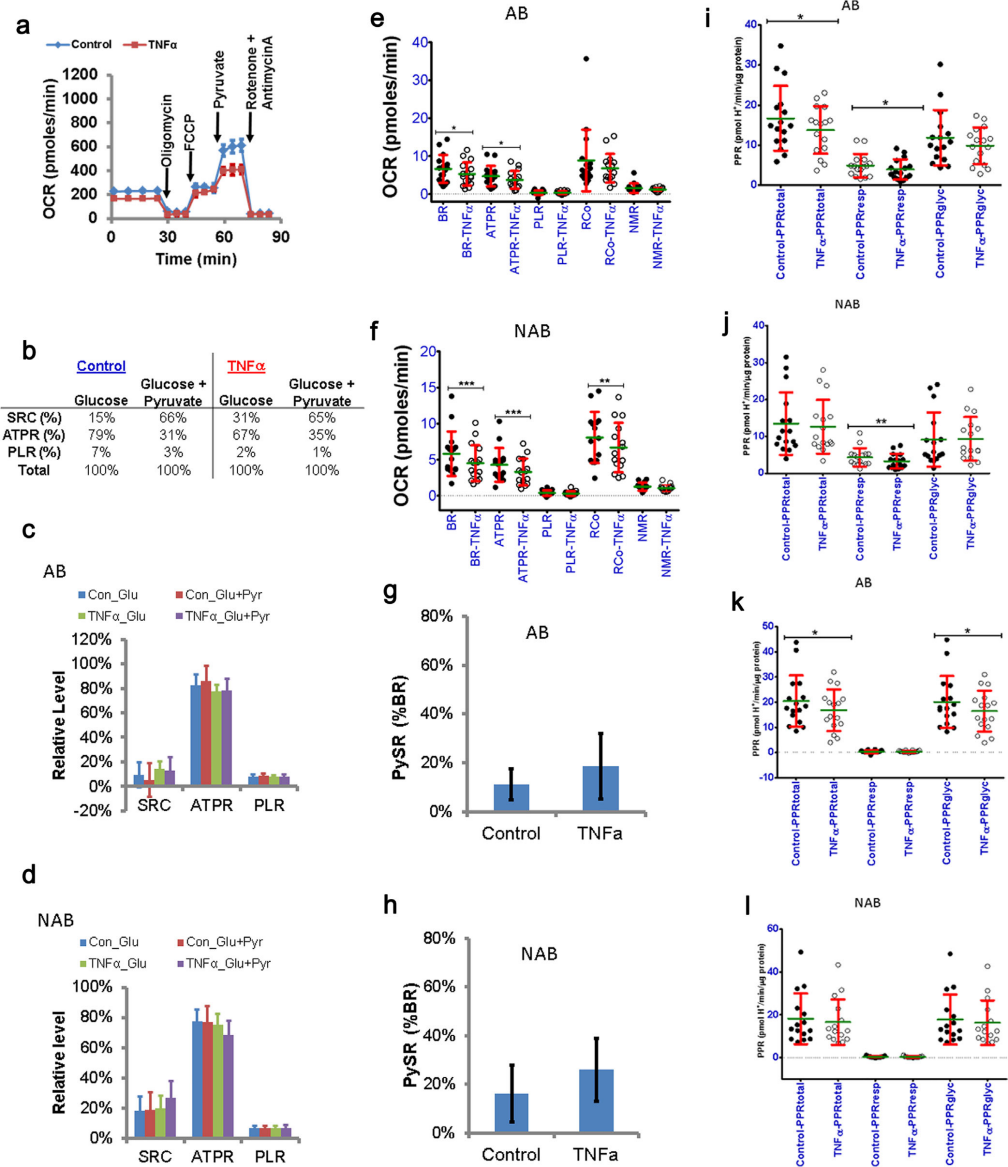
Bioenergetic response of HMECs to TNFα treatment: a) Respiratory profiles of control versus TNFα‐treated SS206 HMECs. b) Effect of TNFα on mitochondrial bioenergetics of SS206 HMECs. Values calculated based on the respiratory profiles in panel A. c) Effect of TNFα on mitochondrial bioenergetics of AB‐HMECs (*n* = 16, mean ± sem). d) Effect of TNFα on mitochondrial bioenergetics of NAB‐HMECs (*n* = 16, mean ± SD, OCR/pmoles/µg protein). e) Effect of TNFα on respiration rates supporting different bioenergetic parameters in AB‐HMECs (*n* = 16, mean ± SD, OCR/pmoles/µg protein, *p ≤ 0.05). f) Effect of TNFα on respiration rates supporting different bioenergetic parameters in NAB‐HMECs (*n* = 16, mean ± SD, OCR/pmoles/µg protein, **p ≤ 0.01, *** ≤ 0.001). g) Effect of TNFα on PySR in AB‐HMECs (*n* = 16, mean ± sem). h) Effect of TNFα on PySR in NAB‐HMECs (*n* = 16, mean ± sem). i) Effect of TNFα on basal PPR in AB‐HMECs (*n* = 16, mean ± SD, Control vs. TNFα *p ≤ 0.05). j) Effect of TNFα on basal PPR in NAB‐HMECs (*n* = 16, mean ± SD, Control vs. TNFα, ** ≤ 0.01). k) Effect of IGF1 on PPR in AB‐HMECs after oligomycin addition (*n* = 16, mean ± SD, Control vs. TNFα *p ≤ 0.05). l) Effect of TNFα on PPR in NAB‐HMECs after oligomycin addition (*n* = 16, mean ± SD, Control vs. TNFα)

TNFα is another host factor that is implicated in breast cancer susceptibility. TNFα promoter polymorphisms are associated with breast cancer risk (Szlosarek et al., 2006). Therefore, we tested TNFα effects on breast epithelial cells bioenergetics. Cells were exposed to TNFα for 24 hr before respirometry. Respirometry profiles of control and TNFα‐treated cells were obtained in side‐by‐side assays as shown for cells from one individual (SS206, Figure 6a). In these cells TNFα decreased respiratory activity. The corresponding values for parameters indicative of mitochondrial bioenergetics, the SRC, ATPR, and PLR are shown in Figure 6b. Differences in control versus TNFα‐treated cells were more apparent on glucose alone versus glucose + pyruvate. This suggests that TNFα alters glucose metabolism of SS206‐HMECs. Overall TNFα did not alter mitochondrial bioenergetics of AB‐ and NAB‐HMECs (Figure 6c,d) despite significant reduction in respiratory activities (Figure 6e,f). The fraction of samples showing PySR^+ve^ increased only in AB‐HMECs from 13% (*n* = 2/16) to 31% (*n* = 5/16, Table 6). It did not change in NAB‐HMECs (31% each control vs. TNFα, Table 6). This suggests that TNFα affects pyruvate metabolism relatively more in epithelial cells from tumor affected breasts compared to non‐affected breasts. However, the mean PySR values in AB and NAB categories did not differ significantly (Figure 6g,h). Together, these data suggest that while TNFα can suppress the respiratory activity of AB‐ and NAB‐HMECs, its effects on parameters indicative of mitochondrial bioenergetics (i.e., SRC, ATPR, and PLR) are minimal and individual‐specific.

**Table 6:** Effect of TNFα on PySR

AB			NAB		
ID	Control	TNFα	ID	Control	TNFα
110.ROH	67%	128%	110.ROH	70%	170%
156.ROH	79%	163%	156.ROH	−21%	5%
178.ROH	−7%	−1%	178.ROH	−19%	−11%
179.ROH	9%	3%	179.ROH	−15%	6%
206.ROH	14%	34%	229.ROH	−4%	13%
207.ROH	7%	14%	231.ROH	−14%	−9%
208.ROH	−5%	−3%	248.ROH	−17%	−15%
218.ROH	1%	−15%	250.ROH	−11%	−3%
219.ROH	−4%	−11%	251.ROH	−12%	−2%
229.ROH	2%	−7%	SS206	146%	108%
231.ROH	−11%	−19%	SS208	85%	54%
240.ROH	−4%	23%	SS212	26%	16%
245.ROH	−7%	−11%	SS213	7%	−5%
248.ROH	−15%	−32%	SS220	35%	77%
250.ROH	−9%	−4%	SS229	−8%	−10%
251.ROH	11%	36%	SS234	15%	24%
n	16	16	n	16	16
Mean	8%	19%	Mean	16%	26%
SD	27%	53%	SD	47%	52%
SE	7%	13%	SE	12%	13%
*p*‐value	0.152		*p*‐value	0.227	

Means of control versus TNFα, treated cells were compared by paired Student's *t*‐test and *p* values are given.

The effects of TNFα on the proton production rate (PPR) in control versus TNFα‐treated cells were compared. In both AB‐ and NAB‐HMECs, TNFα reduced respiratory PPR under basal condition (Figure 6i,j). However, this correlated with significant reduction in total PPR only in AB‐HMECs (Figure 6i). Under oligomycin‐treated condition, TNFα significantly suppressed glycolytic PPR only in AB‐HMECs and it correlated with a reduction in total PPR (Figure 6k). TNFα did not have a notable effect on PPR in oligomycin treated NAB‐HMECs (Figure 6l). Under FCCP‐treated conditions, the reduced total acidification correlated with reduced glycolytic PPR (Table S3). Unlike AB‐HMECs, the respiratory PPR was reduced in FCCP‐treated NAB‐HMECs, and the difference in total PPR was significant only in the presence of exogenous pyruvate. These data suggest that TNFα effect on extracellular acidification was more prominent in AB‐HMECs compared to NAB‐HMECs (Table S3). In terms of percent respiratory and glycolytic PPRs, there were no significant differences between control and TNFα treated cells, which may be attributed to individual‐specific variation (Table S4). These data suggest that TNFα differentially affects metabolism of AB‐ versus NAB‐HMECs. While in AB group glycolysis is affected in NAB group the respiration is more affected. Thus bioenergetic responses of epithelial cells from tumor‐affected and non‐affected breasts are different to TNFα.

### 3.4 Analysis of the IGF1 effects on carbon substrates by metabolic fingerprinting

**Figure 7:**
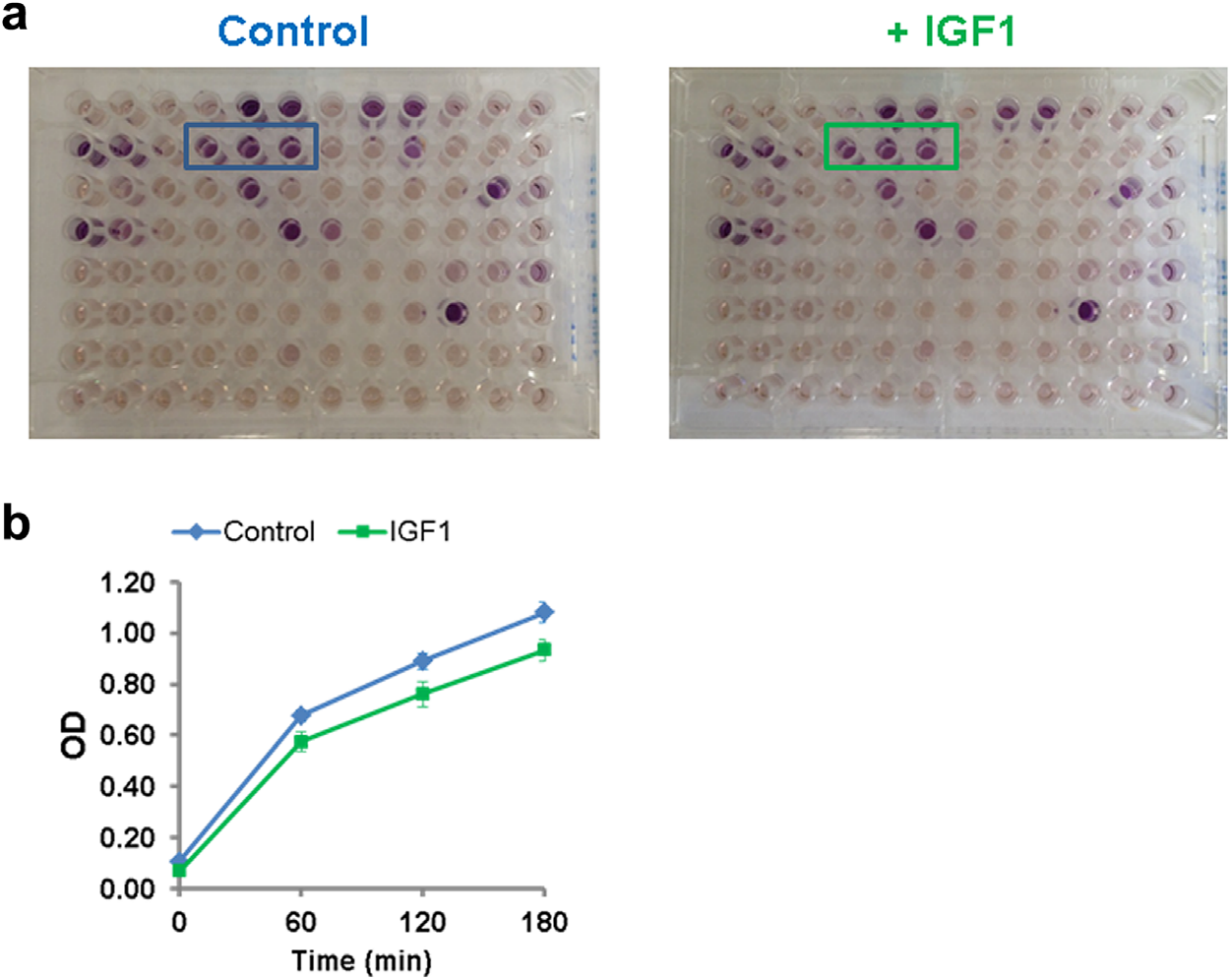
Effect of IGF1 on metabolic phenotype of HMECs. a) A representative PM‐M1 based metabolic fingerprinting of SS212 HMECs. Wells with glucose as substrate are boxed. 10,000 cells were seeded with or without IGF1 in IF1 medium as described in materials and methods. 22 hr later BioLog's redox dye MA was added to monitor color. Picture taken at 24 hr post redox MA addition. b) IGF1 effect on glucose utilization in control and treated cells. Absorbance values from immediately after dye addition to 180 min are plotted

To determine how IGF1 alters carbon substrate utilization in treated cells, we used 96‐well microarrays with different carbon sources PM‐M1 from BioLog Inc. (Hayward, CA, Table S5). Use of individual substrates were scored by monitoring the absorbance of redox dye MA, which develops purple color in the presence of NADH and NADPH without affecting cell viability (Bochner et al., 2011). It does not develop color in the presence of NAD^+^ and NADP^+^ (data not shown, N. Yadava & B. Bochner). Therefore, it informs about overall anabolic and catabolic metabolism of a given substrate indirectly by NADH and NADPH production. Figure 7a shows representative maps for control and IGF1‐treated cells. In IGF1‐treated cells, glucose use was significantly reduced as revealed by reduced absorbance at 590 nm (Figure 7b). We also compared the rate of dye reduction by control and IGF1‐treated HMECs from 6 women within 30–60 min of dye addition. Four of these 6 samples showed 12–31% reduced rate of dye reduction. This suggests that IGF1 reduces glucose‐supported NADH/NADPH production in the majority of HMECs. Together these data suggest that IGF1 can alter glucose use in normal breast epithelial cells, and its response can vary in different individuals.

### 3.5 Effects of IGF1 on radiation‐induced cell death

**Figure 8:**
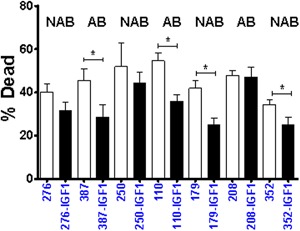
Effect of IGF1 on radiation sensitivity of HMECs. γ‐radiation‐induced death in control and IGF1‐treated HMECs is shown. Open and filled bars represented control and 5 Gy γ‐radiation‐treated cells, respectively. Cell death post‐24 hr radiation is reported. Student's *t*‐test was used to compare the difference between control and IGF1‐treated groups (mean ± sem, *n* = at least four wells/group, *p ≤ 0.05)

To determine the biological relevance of cytokines effects on metabolism of HMECs, we determined radiation‐induced death in control versus IGF1 treated cells. We measured the response of IGF1 on radiation‐induced cell death on a set of randomly selected samples, which consisted of 3 AB and 4 NAB samples. Our data demonstrates that IGF1 significantly suppresses radiation‐induced death in 57% (4/7) of samples, and there was a trend toward a reduction in all but one line. The suppressive effect of IGF1 was observed in both AB and NAB categories. IGF1 reduced basal respiration by 34–64% reduction in 4 samples (110.ROH, 179.ROH, 208.ROH, and 250.ROH). Of these, 208.ROH and 250.ROH were PySR^−ve^ and they did not show a difference in radiation response compared to untreated controls (Figure 8). The AB 110.ROH and NAB 179. ROH were PySR^−ve^ but became PySR^+ve^ following IGF1 treatment. They displayed impaired radiation response. These data suggest that radiation responsiveness can be suppressed by IGF1 treatment in both AB‐ and NAB‐HMECS, and there is inter‐individual variation.

## 4 DISCUSSION

In this study we have quantified inter‐individual difference in bioenergetics of normal/benign breast epithelial cells. Specifically, we have compared the bioenergetics of epithelial cells from tumor‐affected and non‐affected breasts. At individual level, we observed a six‐ to eightfold variation in basal respiratory activity in both AB and NAB groups. Similar variation in extracellular acidification, contributed by glycolysis and respiration, was also noted. While we observed individual‐specific differences in respiratory activity of AB‐ and NAB‐HMECs, the overall mitochondrial bioenergetics of both groups was comparable. In both groups, IGF1 and TNFα reduced respiration and extracellular acidification rates. This study highlights the existence of individual‐specific difference in metabolism, specifically in pyruvate oxidation, of normal breast epithelial cells (Figure 3c). Further, the suppressive effects of IGF1 and TNFα on HMECs metabolism demonstrate how host factors can affect cellular bioenergetics of normal breast epithelial cells (Figures 5 and 6). The pre‐existing metabolic differences in normal breast epithelium and elevated levels of cytokines (e.g., IGF1 and TNFα) may determine susceptibility to oncogenic transformation and thereby increase breast cancer risk in some women. Our observations with breast epithelial cells from cancer patients and women without any cancer history suggest that the PySR^−ve^ phenotype may be associated with susceptibility to breast cancer. Therefore, it will be beneficial to determine the exact frequency of PySR^−ve^ phonotype in healthy women using a larger cohort and find its correlation with breast cancer risk in prospective studies.

The current dogma is that cancer cells reprogram their metabolism to survive and proliferate. The metabolic reprograming is attributed to alterations in oncogenes and tumor suppressors. While this concept underscores the significance of metabolic effects downstream of oncogenes and tumor suppressors in cancer development, it does not inform us about the role of pre‐existing metabolic differences in normal cells that can influence the individual susceptibility to cancer. However, there are clear examples demonstrating that cellular metabolic differences can cause predisposition to tumorigenesis. Hereditary mutations in genes encoding succinate dehydrogenase (SDH) and fumarate hydratase (FH) provide strong evidence for the role of pre‐existing metabolic differences in susceptibility to tumorigenesis (Baysal, Rubinstein, & Taschner, 2001; Evenepoel et al., 2015).

Mitochondrial metabolism can vary from one individual to another. This is because a large number of genes control structure, function, and regulation of the enzymes involved in the TCA cycle and oxidative phosphorylation system, and in the delivery of the substrates to the respiratory chain. Mitochondrial DNA variations and single nucleotide polymorphisms (SNPs) are found in numerous nuclear genes relating to mitochondrial metabolism (https://www.ncbi.nlm.nih.gov/snp). In addition to genetic factors, mitochondrial metabolism can influence carcinogenesis either in isolation or in association with host and environmental factors. Thus, the quantification of individual‐specific metabolic differences and identification of the factors that alter metabolism are necessary to understand their impact on cancer risk. This study is a step in the direction toward quantifying individual‐specific variation in breast epithelial cells metabolism. It supports a potential role of pre‐existing differences in oxidative metabolism to breast cancer risk. Our data show several fold variation in respiratory activities of breast epithelial cells from different women (Figure 4). Pyruvate‐stimulated respiration (PySR) is a key bioenergetic parameter to assess individual‐ specific variation. Our observation that there is a difference in exogenous pyruvate oxidation in cells from different women is interesting. In particular, the observation that cells from cancer patients are mostly PySR^−ve^, suggests that this phenotype could be linked with breast cancer risk (Figure 3c–e). The analysis of proton production rates in IGF1 and TNFα‐treated cells revealed differences in metabolism of normal epithelial cells from tumor‐affected versus non‐affected breasts. The cytokines effect on acidification was more prominent in cells from affected breasts (Figures 5i–l and 6i–l). This is relevant to autocrine tumors, which may secrete cytokines that can alter their own metabolism as well as neighboring cells. As in this study, we have not assessed the PySR^−ve/+ve^ phenotype of matching tumor cells; it is not possible to predict the phenotype after oncogenic transformation. If oncogenically transformed cells retain the PySR^−ve^ phenotype, then they will not be able to use exogenous pyruvate for bioenergetics as suggested by the recently proposed “*Reverse Warburg hypothesis*” (Pavlides et al., 2009). They can only use exogenous pyruvate if they secrete cytokines such as IGF1 locally. The suppression of oxidative metabolism in IGF1‐ and TNFα‐treated cells is suggested by both respiration and acidification analyses. Therefore, irrespective of whether transformed cells respire their oxidative metabolism is not normal. Our follow‐up studies will focus comparing bioenergetics of normal breast epithelial cells before and after oncogenic transformation from different women.

Cells from individual donors may differ in many respects. One of these is differences in metabolism as revealed by this study. Parity and genetic background may play a role in individual differences. Differences in cell populations could also be a factor to consider (Linnemann et al., 2015). Thus there is a possibility that the percentage of basal to luminal cells differ in the early passage HMECs from women with cancer versus without cancer. Such variation in HMECs preparation may contribute to noted individual‐specific metabolic differences. Thus, future studies looking at purified subpopulations may be required to tease out this potential confounding factor before deciphering the role of genetic variations in nuclear and mitochondrial components of the oxidative metabolism.

In most cells, glucose is the primary fuel as well as the carbon source for biosynthesis. If glucose is completely oxidized for ATP synthesis, then all its carbons will be released as CO_2_. Therefore, cells balance the use of glucose carbon for bioenergetics versus biosynthesis. At the center of this balance lies the pyruvate metabolism that connects all three aspects of metabolic reprograming, that is, bioenergetics, redox balance, and biosynthesis. Cytosolic NAD^+^ regeneration from NADH depends on pyruvate to lactate conversion, NADH redox shuttles, and its oxidation inside mitochondria to support respiratory chain function. Changes in NAD^+^/NADH redox within cytosol and mitochondria can alter pyruvate production, secretion, and oxidation (Titov et al., 2016). Cells severely defective in respiratory chain function are dependent on exogenous pyruvate (King & Attardi, 1989) or asparagine/aspartate (Birsoy et al., 2015; Ditta, Soderberg, Landy, & Scheffler, 1976; Sullivan et al., 2015). Thus respiratory chain is functionally linked with synthesis of nonessential amino acids such as aspartate, and it influences proliferation. The pyruvate oxidation inside mitochondria can be suppressed by multiple mechanisms such as reduced production by glycolysis, conversion to lactate and other metabolites, excretion, suppressed transport into mitochondria, and reduced oxidation by the TCA cycle. Mitochondrial pyruvate carriers MPC1 and MPC2 reside in the inner mitochondrial membrane (Bricker et al., 2012; Herzig et al., 2012). MPC1 is often lost in cancer cells, and its expression is linked with anti‐proliferative phenotype (Schell et al., 2014). The cells with reduced MPCs rely on glutamine anaplerosis to feed the TCA cycle without any difference in glucose and glutamine uptake. They also secret pyruvate and reprogram metabolism to promote lipid synthesis and branched chain amino acids oxidation. In many cancers the PK‐M2 isoform, which is negatively regulated by post‐translational modifications via growth factor signaling, is four‐ to six‐fold higher than the PK‐M1 isoform. Thus cells expressing PK‐M2 isoform can limit the production of pyruvate and divert it away from oxidation to support biosynthesis. In PySR^+ve^ cells either pyruvate production from glucose is reduced or pyruvate is diverted away from the oxidation. Because exogenous pyruvate increases respiration, a low mitochondrial pyruvate carrier is not expected in PySR^+ve^ cells for the limitations in the respiratory capacity on glucose as sole fuel. On the other hand, in PySR^−ve^ cells glucose itself is able to support maximal respiratory capacity. That means the cells are also not limited in mitochondrial pyruvate carrier.

Alterations in pyruvate oxidation have been linked with susceptibility to oncogene‐induced senescence, a protective mechanism against tumorigenesis (Kaplon et al., 2013). Therefore the observation of difference in pyruvate oxidation in cells from women with and without cancer is highly relevant to breast cancer risk. One of the potential mechanisms for breast cancer susceptibility due to *BRCA1* mutations is by alterations in cellular metabolism. A recent study suggests that *BRCA1* haploinsufficiency (*BRCA1^+/^* ^*−*^) decreases intracellular pyruvate by 78% and favors biosynthesis of lipids and branched‐chain amino acids (Cuyas et al., 2016). The exit of pyruvate as citrate for lipid synthesis could be a potential cause for reduced intracellular pyruvate. The PySR^−ve/+ve^ state of *BRCA1^+/−^* cells is unknown. These cells may be PySR^−ve^ if exogenous pyruvate will be directed toward anabolism without producing a significant difference in respiratory response. It may also result in altered glutamine metabolism. Decreased pyruvate flux in mitochondria is linked with increased glutamine use (Le et al., 2012; Metallo et al., 2011).

The difference in exogenous pyruvate oxidation and respiratory capacity of cells clearly indicates variation in mitochondrial metabolism. This can involve oxidative metabolism at the levels of physical contents of the complexes of oxidative phosphorylation system, the TCA cycle, and redox metabolism. The impairments of oxidative metabolism can impair signaling pathways that promote tumorigenesis (Yadava, Schneider, Jerry, & Kim, 2013). Respiratory chain impairments suppress tumor suppressor protein p53 and provide protection against radiation‐induced death (Compton et al., 2011). Respiratory chain impairments also genetically inactivate p53 in neural stem cells (Bartesaghi et al., 2015). P53 is also connected to mitochondria by estrogen signaling, which plays a key role in breast tumorigenesis (Wickramasekera & Das, 2014). Since p53 pathway is the most potent tumor suppressing pathway, its suppression can predispose to breast tumorigenesis in epithelial cells with compromised respiratory activity. Mitochondrial metabolism is also linked with suppression of tumorigenic property of metastatic cells (Kaipparettu et al., 2013). Because pyruvate metabolism influences oncogene‐induced senescence, a protective mechanism against tumorigenesis (Kaplon et al., 2013), the pre‐existing differences in pyruvate metabolism is relevant to breast cancer susceptibility.

In summary, our study supports the existence of inter‐individual variation in cellular bioenergetics. This is primarily reflected in respiratory activity and its response to exogenous pyruvate. The difference in exogenous pyruvate oxidation detected as pyruvate‐stimulated respiration (PySR) is an interesting finding of the metabolic differences present in normal breast epithelial cells from different women. Based on the relative frequency of the PySR^−ve^ phenotype between cells from cancer patients and women without any cancer history, we predict that PySR^−ve^ phenotype is linked with breast cancer risk. Furthermore, cytokines such as IGF1 and TNFα can alter mammary epithelial cells bioenergetics as host factors. The response to cytokines is also variable among breast epithelial cells from different women. Together, the bioenergetic variation and its response to cytokines may alter susceptibility to oncogenic transformation.

## Supporting information

Additional Supporting Information may be found online in the supporting information tab for this article.

Supporting Table S1.

Supporting Table S2.

Supporting Table S3.

Supporting Table S4.

Supporting Table S5.

## References

[jcp25932-bib-0001] Bartesaghi, S. , Graziano, V. , Galavotti, S. , Henriquez, N. V. , Betts, J. , Saxena, J. , … Salomoni, P. (2015). Inhibition of oxidative metabolism leads to p53 genetic inactivation and transformation in neural stem cells. Proceedings of the National Academy of Sciences of the United States of America, 112(4), 1059–1064. 25583481 10.1073/pnas.1413165112 PMC4313844

[jcp25932-bib-0002] Baysal, B. E. , Rubinstein, W. S. , & Taschner, P. E. (2001). Phenotypic dichotomy in mitochondrial complex II genetic disorders. Journal of Molecular Medicine (Berlin, Germany), 79(9), 495–503. 10.1007/s001090100267 11692162

[jcp25932-bib-0003] Birsoy, K. , Wang, T. , Chen, W. W. , Freinkman, E. , Abu‐Remaileh, M. , & Sabatini, D. M. (2015). An essential role of the mitochondrial electron transport chain in cell proliferation is to enable aspartate synthesis. Cell, 162(3), 540–551. 26232224 10.1016/j.cell.2015.07.016 PMC4522279

[jcp25932-bib-0004] Bochner, B. R. , Siri, M. , Huang, R. H. , Noble, S. , Lei, X. H. , Clemons, P. A. , & Wagner, B. K. (2011). Assay of the multiple energy‐producing pathways of mammalian cells. PLoS ONE, 6(3), e18147. 21455318 10.1371/journal.pone.0018147 PMC3063803

[jcp25932-bib-0005] Bricker, D. K. , Taylor, E. B. , Schell, J. C. , Orsak, T. , Boutron, A. , Chen, Y. C. , … Rutter, J. (2012). A mitochondrial pyruvate carrier required for pyruvate uptake in yeast, Drosophila, and humans. Science (New York, NY), 337(6090), 96–100. 10.1126/science.1218099 PMC3690818 22628558

[jcp25932-bib-0006] Compton, S. , Kim, C. , Griner, N. B. , Potluri, P. , Scheffler, I. E. , Sen, S. , … Yadava, N. (2011). Mitochondrial dysfunction impairs tumor suppressor p53 expression/function. The Journal of Biological Chemistry, 286(23), 20297–20312. 21502317 10.1074/jbc.M110.163063 PMC3121470

[jcp25932-bib-0007] Cuyas, E. , Fernandez‐Arroyo, S. , Alarcon, T. , Lupu, R. , Joven, J. , & Menendez, J. A. (2016). Germline BRCA1 mutation reprograms breast epithelial cell metabolism towards mitochondrial‐ dependent biosynthesis: Evidence for metformin‐based “starvation“ strategies in BRCA1 carriers. Oncotarget, 7(33), 52974–52992. 27259235 10.18632/oncotarget.9732 PMC5288162

[jcp25932-bib-0008] DeBerardinis, R. J. , & Chandel, N. S. (2016). Fundamentals of cancer metabolism. Science Advances, 2(5), e1600200. 27386546 10.1126/sciadv.1600200 PMC4928883

[jcp25932-bib-0009] Ditta, G. , Soderberg, K. , Landy, F. , & Scheffler, I. E. (1976). The selection of Chinese hamster cells deficient in oxidative energy metabolism. Somatic Cell Genetics, 2(4), 331–344. 1027147 10.1007/BF01538838

[jcp25932-bib-0010] Evenepoel, L. , Papathomas, T. G. , Krol, N. , Korpershoek, E. , de Krijger, R. R. , Persu, A. , & Dinjens, W. N. (2015). Toward an improved definition of the genetic and tumor spectrum associated with SDH germ‐line mutations. Genetics in Medicine, 17(8), 610–620. 25394176 10.1038/gim.2014.162

[jcp25932-bib-0011] Gerencser, A. A. , Neilson, A. , Choi, S. W. , Edman, U. , Yadava, N. , Oh, R. J. , … Brand, M. D. (2009). Quantitative microplate‐based respirometry with correction for oxygen diffusion. Analytical Chemistry, 81(16), 6868–6878. 19555051 10.1021/ac900881z PMC2727168

[jcp25932-bib-0012] Herzig, S. , Raemy, E. , Montessuit, S. , Veuthey, J. L. , Zamboni, N. , Westermann, B. , … Martinou, J. C. (2012). Identification and functional expression of the mitochondrial pyruvate carrier. Science (New York, NY), 337(6090), 93–96. 10.1126/science.1218530 22628554

[jcp25932-bib-0013] Jekabsons, M. B. , & Nicholls, D. G. (2004). In situ respiration and bioenergetic status of mitochondria in primary cerebellar granule neuronal cultures exposed continuously to glutamate. The Journal of Biological Chemistry, 279(31), 32989–33000. 15166243 10.1074/jbc.M401540200

[jcp25932-bib-0014] Kaaks, R. , Johnson, T. , Tikk, K. , Sookthai, D. , Tjonneland, A. , Roswall, N. , … Lukanova, A. (2014). Insulin‐like growth factor I and risk of breast cancer by age and hormone receptor status‐A prospective study within the EPIC cohort. International Journal of Cancer, 134(11), 2683–2690. 24248481 10.1002/ijc.28589

[jcp25932-bib-0015] Kaipparettu, B. A. , Ma, Y. , Park, J. H. , Lee, T. L. , Zhang, Y. , Yotnda, P. , … Wong, L. J. (2013). Crosstalk from non‐cancerous mitochondria can inhibit tumor properties of metastatic cells by suppressing oncogenic pathways. PLoS ONE, 8(5), e61747. 23671572 10.1371/journal.pone.0061747 PMC3650012

[jcp25932-bib-0016] Kaplon, J. , Zheng, L. , Meissl, K. , Chaneton, B. , Selivanov, V. A. , Mackay, G. , … Peeper, D. S. (2013). A key role for mitochondrial gatekeeper pyruvate dehydrogenase in oncogene‐induced senescence. Nature, 498(7452), 109–112. 23685455 10.1038/nature12154

[jcp25932-bib-0017] Kim, C. , Patel, P. , Gouvin, L. M. , Brown, M. L. , Khalil, A. , Henchey, E. M. , … Yadava, N. (2014). Comparative analysis of the mitochondrial physiology of pancreatic beta cells. Cell Death & Disease, 3(1), 110. 10.4172/2167-7662.1000110 PMC4190029 25309834

[jcp25932-bib-0018] King, M. P. , & Attardi, G. (1989). Human cells lacking mtDNA: Repopulation with exogenous mitochondria by complementation. Science (New York, NY), 246(4929), 500–503. 10.1126/science.2814477 2814477

[jcp25932-bib-0019] Le, A. , Lane, A. N. , Hamaker, M. , Bose, S. , Gouw, A. , Barbi, J. , … Dang, C. V. (2012). Glucose‐independent glutamine metabolism via TCA cycling for proliferation and survival in B cells. Cell Metabolism, 15(1), 110–121. 22225880 10.1016/j.cmet.2011.12.009 PMC3345194

[jcp25932-bib-0020] Linnemann, J. R. , Miura, H. , Meixner, L. K. , Irmler, M. , Kloos, U. J. , Hirschi, B. , … Scheel, C. H. (2015). Quantification of regenerative potential in primary human mammary epithelial cells. Development, 142(18), 3239–3251. 26071498 10.1242/dev.123554 PMC4582177

[jcp25932-bib-0021] Metallo, C. M. , Gameiro, P. A. , Bell, E. L. , Mattaini, K. R. , Yang, J. , Hiller, K. , … Stephanopoulos, G. (2011). Reductive glutamine metabolism by IDH1 mediates lipogenesis under hypoxia. Nature, 481(7381), 380–384. 22101433 10.1038/nature10602 PMC3710581

[jcp25932-bib-0022] Mookerjee, S. A. , & Brand, M. D. (2015). Measurement and analysis of extracellular acid production to determine glycolytic rate. Journal of Visualized Experiments, (106), e53464. 26709455 10.3791/53464 PMC4692795

[jcp25932-bib-0023] Mookerjee, S. A. , Goncalves, R. L. , Gerencser, A. A. , Nicholls, D. G. , & Brand, M. D. (2015). The contributions of respiration and glycolysis to extracellular acid production. Biochimica Et Biophysica Acta, 1847(2), 171–181. 25449966 10.1016/j.bbabio.2014.10.005

[jcp25932-bib-0024] Mookerjee, S. A. , Nicholls, D. G. , & Brand, M. D. (2016). Determining maximum glycolytic capacity using extracellular flux measurements. PLoS ONE, 11(3), e0152016. 27031845 10.1371/journal.pone.0152016 PMC4816457

[jcp25932-bib-0025] Pavlides, S. , Whitaker‐Menezes, D. , Castello‐Cros, R. , Flomenberg, N. , Witkiewicz, A. K. , Frank, P. G. , … Lisanti, M. P. (2009). The reverse Warburg effect: Aerobic glycolysis in cancer associated fibroblasts and the tumor stroma. Cell Cycle (Georgetown, Tex), 8(23), 3984–4001. 10.4161/cc.8.23.10238 19923890

[jcp25932-bib-0026] Schell, J. C. , Olson, K. A. , Jiang, L. , Hawkins, A. J. , Van Vranken, J. G. , Xie, J. , … Rutter, J. (2014). A role for the mitochondrial pyruvate carrier as a repressor of the Warburg effect and colon cancer cell growth. Molecular Cell, 56(3), 400–413. 25458841 10.1016/j.molcel.2014.09.026 PMC4268416

[jcp25932-bib-0027] Suhre, K. , Shin, S. Y. , Petersen, A. K. , Mohney, R. P. , Meredith, D. , Wagele, B. , … Gieger, C. (2011). Human metabolic individuality in biomedical and pharmaceutical research. Nature, 477(7362), 54–60. 21886157 10.1038/nature10354 PMC3832838

[jcp25932-bib-0028] Suhre, K. , Wallaschofski, H. , Raffler, J. , Friedrich, N. , Haring, R. , Michael, K. , … Nauck, M. (2011). A genome‐wide association study of metabolic traits in human urine. Nature Genetics, 43(6), 565–569. 21572414 10.1038/ng.837

[jcp25932-bib-0029] Sullivan, L. B. , Gui, D. Y. , Hosios, A. M. , Bush, L. N. , Freinkman, E. , & Vander Heiden, M. G. (2015). Supporting aspartate biosynthesis is an essential function of respiration in proliferating cells. Cell, 162(3), 552–563. 26232225 10.1016/j.cell.2015.07.017 PMC4522278

[jcp25932-bib-0030] Szlosarek, P. , Charles, K. A. , & Balkwill, F. R. (2006). Tumour necrosis factor‐alpha as a tumour promoter. European Journal of Cancer, 42(6), 745–750. 16517151 10.1016/j.ejca.2006.01.012

[jcp25932-bib-0031] Titov, D. V. , Cracan, V. , Goodman, R. P. , Peng, J. , Grabarek, Z. , & Mootha, V. K. (2016). Complementation of mitochondrial electron transport chain by manipulation of the NAD+/NADH ratio. Science (New York, NY), 352(6282), 231–235. 10.1126/science.aad4017 PMC4850741 27124460

[jcp25932-bib-0032] To, S. Q. , Knower, K. C. , & Clyne, C. D. (2013). Origins and actions of tumor necrosis factor alpha in postmenopausal breast cancer. Journal of Interferon & Cytokine Research, 33(7), 335–345. 23472660 10.1089/jir.2012.0155

[jcp25932-bib-0033] Wallace, D. C. (2005). A mitochondrial paradigm of metabolic and degenerative diseases, aging, and cancer: A dawn for evolutionary medicine. Annual Review of Genetics, 39, 359–407. 10.1146/annurev.genet.39.110304.095751 PMC2821041 16285865

[jcp25932-bib-0034] Wallace, D. C. (2013). A mitochondrial bioenergetic etiology of disease. The Journal of Clinical Investigation, 123(4), 1405–1412. 23543062 10.1172/JCI61398 PMC3614529

[jcp25932-bib-0035] Wickramasekera, N. T. , & Das, G. M. (2014). Tumor suppressor p53 and estrogen receptors in nuclear‐ mitochondrial communication. Mitochondrion, 16, 26–37. 24177747 10.1016/j.mito.2013.10.002 PMC4026364

[jcp25932-bib-0036] Wu, M. , Neilson, A. , Swift, A. L. , Moran, R. , Tamagnine, J. , Parslow, D. , … Ferrick, D. A. (2007). Multiparameter metabolic analysis reveals a close link between attenuated mitochondrial bioenergetic function and enhanced glycolysis dependency in human tumor cells. American Journal of Physiology Cell Physiology, 292(1), C125–C136. 16971499 10.1152/ajpcell.00247.2006

[jcp25932-bib-0037] Yadava, N. , Schneider, S. S. , Jerry, D. J. , & Kim, C. (2013). Impaired mitochondrial metabolism and mammary carcinogenesis. Journal of Mammary Gland Biology and Neoplasia, 18(1), 75–87. 23269521 10.1007/s10911-012-9271-3 PMC3581737

